# Phenethylisothiocyanate Alters Site- and Promoter-Specific Histone Tail Modifications in Cancer Cells

**DOI:** 10.1371/journal.pone.0064535

**Published:** 2013-05-28

**Authors:** Yi Liu, Suvobrata Chakravarty, Moul Dey

**Affiliations:** 1 Department of Health & Nutritional Sciences, South Dakota State University, Brookings, South Dakota, United States of America; 2 Department of Chemistry and Biochemistry, South Dakota State University, Brookings, South Dakota, United States of America; Florida International University, United States of America

## Abstract

Site-specific histone modifications are important epigenetic regulators of gene expression. As deregulation of genes often results in complex disorders, corrective modulation of site-specific histone marks could be a powerful therapeutic or disease-preventive strategy. However, such modulation by dietary compounds and the resulting impact on disease risk remain relatively unexplored. Here we examined phenethylisothiocyanate (PEITC), a common dietary compound derived from cruciferous vegetables with known chemopreventive properties under experimental conditions, as a possible modulator of histone modifications in human colon cancer cells. The present study reports novel, dynamic, site-specific chemical changes to histone H3 in a gene-promoter-specific manner, associated with PEITC exposure in human colon tumor-derived SW480 epithelial cells. In addition, PEITC attenuated cell proliferation in a concentration- and time-dependent manner, likely mediated by caspase-dependent apoptotic signalling. The effects of PEITC on histone modifications and gene expression changes were achieved at low, non-cytotoxic concentrations, in contrast to the higher concentrations necessary to halt cancer cell proliferation. Increased understanding of specific epigenetic alterations by dietary compounds may provide improved chemopreventive strategies for reducing the healthcare burden of cancer and other human diseases.

## Introduction

Cancer remains the second-leading cause of all deaths in the United States [1]. Fortunately, many dietary compounds can potently modulate various molecular targets, leading to prevention of cancer initiation, promotion, and progression. In particular, fruits and vegetables are rich sources of biologically active compounds that often have low toxicities but significant efficacies [2]. In the past, cancer was narrowly conceived as a disease of mutations, but newer research also associates the diseased state with the perturbation of cellular regulatory networks, and the disruption of gene function and gene regulation are now both recognized as hallmarks of cancer [3], [4], [5]. Hence, disease-preventive measures aiming to target key elements of the networks regulating gene function, such as chromatin, might be effective. The alterations of site-specific chromatin modifications, known as epigenetic changes, are relevant to clinical oncology, as they are closely associated with gene expression and network perturbations in the diseased state [6], [7]. Therefore, elucidating the role of dietary compounds in resetting the aberrant epigenetic landscapes responsible for altered gene expression may facilitate preventive medical practices.

The epigenetic basis of gene regulation is manifested at the structural unit of chromatin, the nucleosome, which is an assembly of histone octamers wrapped by genomic DNA. Modifications of histones constitute a major molecular control point in the regulation of gene expression, and these modifications are frequently altered in cancers [6], [7]. Among many known histone amino acid tail modifications, methylation and acetylation of the lysine residues on histone H3 have been extensively studied with regard to gene silencing and gene regulation. Dimethylation of H3 at lysine 9 (H3K9me2) and trimethylation of H3 at lysine 27 (H3K27me3) are frequently associated with transcriptional repression and gene silencing [8]. Site-specific histone lysine methylations are catalyzed by histone methyl transferases (HMTs), and the removal of methyl groups are catalyzed by demethylases. Similarly, deacetylation of histones at gene promoters catalyzed by histone deacetylases (HDACs) is correlated with the condensation of chromosomal domains marking regions of transcriptional incompetence and down-regulation of the associated genes [9]. Though in vitro studies of the role of dietary phytochemicals in modulating the levels of HMTs and HDACs exist in small numbers [10], the modulation of position-specific H3 lysine modifications by dietary compounds in a gene-specific manner remains relatively unexplored [11]. Here, we investigated H3-acetylation (H3-Ac) and site-specific H3 lysine methylations (H3K27me3 and H3K9me2) in association with phenethylisothiocyanate (PEITC)-mediated gene expression modulation in human colon cancer cells. This is a follow up of our previous reports on PEITC as a dietary compound with potential anti-inflammatory functions in various experimental models [12], [13].

PEITC occurs naturally in the form of its glucosinolate precursor, gluconasturtiin, in vegetables such as cabbage, cauliflower, wintercress, and broccoli. PEITC has shown potential antioxidant and chemopreventive activity in experimental models of various cancers [14], [15]. It exhibited no apparent toxicity in drug safety studies [16] and is currently in clinical trials for lung cancer treatments (clinicaltrials.gov: NCT00005883, NCT00691132). In mouse, we previously demonstrated that PEITC attenuates colon inflammation and modulates a number of potential biomarkers related to inflammation and colon carcinogenesis. These biomarkers included genes related to the inflammatory response, apoptosis, cell cycle regulation, proliferation, cytokine/chemokine activity, and transcriptional regulation [12], [13].

Colorectal cancer is the second-leading cause of cancer-related deaths in the United States [21]. Interestingly, a mechanistic association between chronic inflammation and an increased risk of cancer arising from inflammation-induced genetic and epigenetic instability is now well accepted [17]. Key players in this association include transcription factors, such as nuclear factor kappa B (NFκB) and signal transducers and activators of transcription (STATs), cytokines/chemokines, and matrix metalloproteinases (MMPs), a multigene family of zinc-dependent extracellular matrix-remodeling endopeptidases. These cellular mediators, some of which we studied previously in mouse models [12], [13], have important functions related to the bypassing of adaptive immunity, proliferation, survival of malignant cells, tumor growth, angiogenesis, invasion, and metastasis [17], [18], [19], [20]. The current study investigated chromatin changes in association with PEITC-mediated modulation of the expression of these mediators in human colon cancer cells.

## Materials and Methods

### Drug and Chemicals

Dimethyl sulfoxide (DMSO), lipopolysaccharide (LPS, from *Escherichia coli* strain O55:B5), penicillin/streptomycin, and phenethylisothiocyanate (PEITC) were purchased from Sigma-Aldrich (St. Louis, MO). Dulbecco’s Modified Eagle’s Medium (DMEM), fetal bovine serum (FBS), and TrypLE were purchased from Gibco (Grand Island, NY). Human interferon-γ (IFNγ) was purchased from R&D Systems (Minneapolis, MN). Phosphate-buffered saline (PBS) was purchased from Thermo Scientific (Rockford, IL). For western blotting, an antibody against β-actin was purchased from Santa Cruz Biotechnology (Santa Cruz, CA), and antibodies against STAT1 and phosphorylated STAT1 (Ser727) were purchased from Millipore (Billerica, MA). Dylight 800 anti-rabbit secondary antibody was purchased from Li-Cor Biosciences (Lincoln, NE). Antibodies used for the chromatin immunoprecipitation (ChIP) assay were anti-acetyl-Histone H3, anti-trimethyl-Histone H3 (Lys27), and rabbit IgG for negative control samples from Upstate Biotechnology (Billerica, MA) and anti-dimethyl-Histone H3 (Lys9) from Abcam (Cambridge, MA). Enzymes used for the ChIP assay were micrococcal nuclease (MNase) from Cell Signaling (Beverly, MA) and Proteinase K from Thermo Scientific Pierce (Rockford, IL). Oligonucleotides were synthesized by IDT DNA Inc. (Coralville, IA). The ChIP assay chemicals aprotinin, DL-1, 4-dithiothreitol (DTT), and Nonidet-P40 (NP-40) were purchased from Thermo Scientific (Rockford, IL), while sodium butyrate, protein-A sepharose, and sucrose were purchased from Sigma-Aldrich (St. Louis, MO). ChIP incubation buffer was purchased from Abcam (Cambridge, MA), and a DNA-purifying slurry for ChIP-grade DNA purification was purchased from Diagenode (Denville, NJ).

### Cell Culture

SW480 (ATCC CCL-228), HT-29 (ATCC HTB-38), and THP-1 (ATCC TIB-202) cell lines obtained from American Type Culture Collection (Manassas, VA) were grown in DMEM supplemented with 10% FBS, 1% penicillin (25 U/ml)/streptomycin (25 µg/ml) in a 95% air/5% CO_2_-humidified atmosphere at 37°C. Three cell lines were cultured as described [24]. Briefly, THP-1 cells were treated with PEITC at a pre-determined dose for 8 h before elicitation with LPS (1 µg/ml). SW480 and HT-29 cells were primed with IFNγ (10 ng/ml) for 12 h, treated with PEITC for 5 h, and then stimulated with LPS (10 or 50 ng/ml) for 4 or 1 h, respectively. LPS and PEITC were dissolved in DMEM and DMSO, respectively, with DMSO used as vehicle. For every experiment, one positive control (cells treated with DMSO and LPS/IFNγ) and one negative control (cells treated with DMSO only) were included. Two replicates were made for each treatment. PEITC treatments were performed at 2.5, 5, 10, and 15 µM concentrations. For analyzing the time-dependent expression changes of STAT1 and other genes of interest (GoI) in response to PEITC, SW480 cells were treated with 10 µM PEITC for 5, 8, 12, and 18 h. All experiments were repeated a minimum of three times.

### Cell Viability Assay, Dose Range Determination, and Cell Proliferation

**Figure 1:**
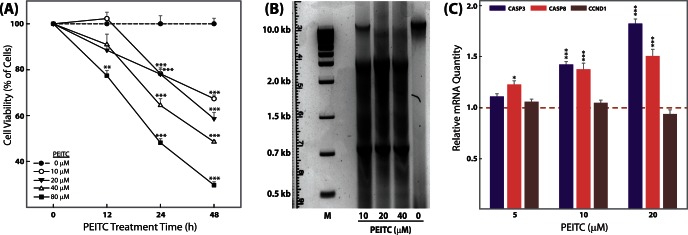
Time- and concentration-dependent effects of PEITC on SW480 cell proliferation. Following 12, 24, and 48 h PEITC exposure and a subsequent 2 h incubation with CellTiter aqueous reagent (Promega), A_490 nm_ was measured in SW480 cells in a BioTek Synergy H4 reader. Percentage cell viability is shown relative to control (no PEITC) as the mean ± SEM (A); Concentration-dependent effects of PEITC at 48 h on DNA fragmentation (B); Concentration-dependent modulation of the expression of genes related to apoptosis and the cell cycle by PEITC at 48 h. GAPDH was used as a housekeeping control for relative quantification of gene expression changes (C); for all experiments n = 3. *p<0.05, **p<0.01, ***p<0.001. No loss in cell viability in the 10 µM PEITC group compared with the no-PEITC group was observed up to 12 h.

**Figure 2:**
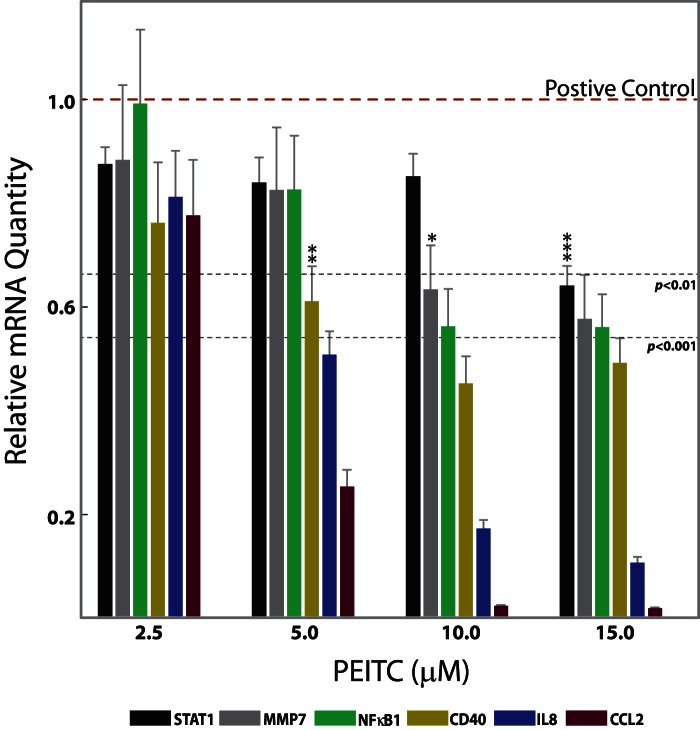
PEITC concentration-dependent mRNA expression of genes of interest (GoI) in SW480 cells at 5 h of PEITC exposure. Six genes are shown for which PEITC-associated changes in H3 modifications were also observed. GoIs for which PEITC-exposure-associated H3 changes were not observed are shown in Table 3, but not in the current figure. The effects of PEITC treatments were measured by the relative mRNA quantity expressed by GoI in the treated cells. The mRNA quantity was measured using real-time RT-PCR, with GAPDH as an internal control. Lower values represent greater inhibitory effects. Values are mean ± SEM (n = 6). *p<0.05, **p<0.01, ***p<0.001. When all values below the dotted line correspond to the same category of *p* value, individual stars are omitted to avoid overcrowding of the figure. Statistical significances were measured relative to the positive control, which are cells treated with LPS and no PEITC, showing the highest gene induction levels.

**Figure 3:**
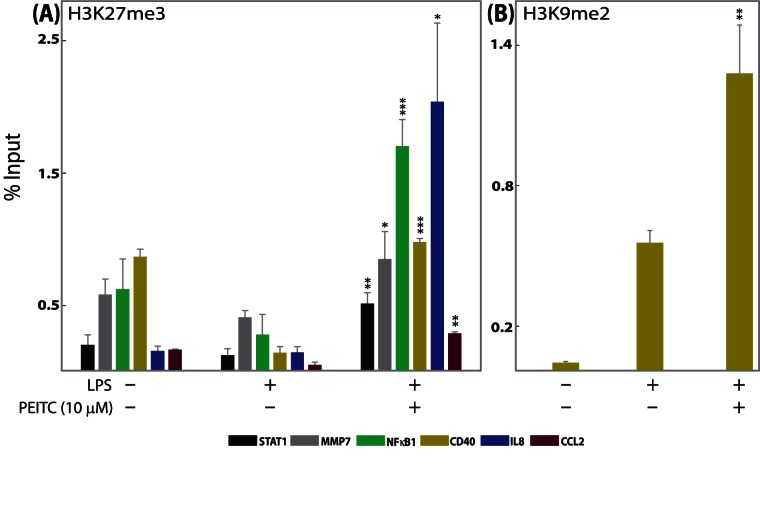
**Histone H3 methylation changes at the promoter regions of genes of interest (GoI) in SW480 cells.** Chromatin from SW480 cells was harvested after **5 h of 10-µM** PEITC treatment. The results of ChIP analyses using (A) anti-trimethyl-Histone H3 Lys27 (H3K27me3) and (B) anti-dimethyl-Histone H3 Lys9 (H3K9me2) antibodies are shown. Out of 13 GoIs tested in SW480 cells, six genes showed statistically significant PEITC-associated changes in H3K27me3 states and one gene showed statistically significant PEITC-associated changes in H3K9me2 state. DNA sequences were quantified by real-time PCR using promoter primers. Average percentage input ± SEM (n = 3) from each experiment is plotted. *p<0.05, **p<0.01, ***p<0.001 compared with positive-control cells.

**Figure 4:**
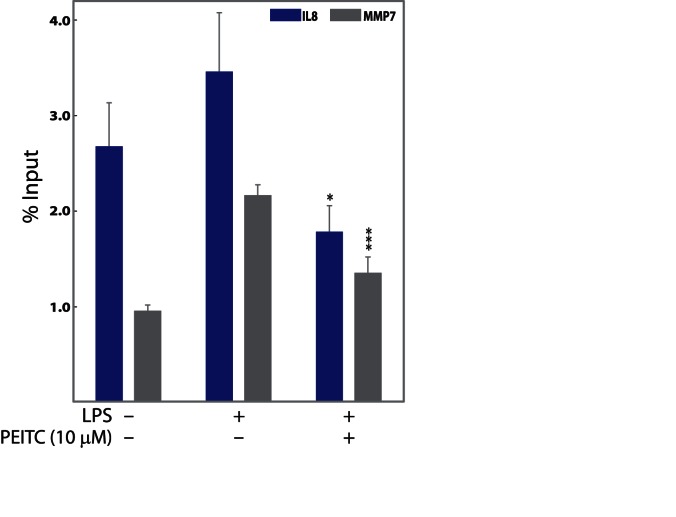
**Histone H3 acetylation changes at the promoter region of genes of interest (GoI) in SW480 cells.** Chromatin from SW480 cells was harvested after 5 h of 10 µM PEITC treatment. Out of 13 GoIs tested in SW480 cells, two genes showed statistically significant PEITC-associated changes in H3-Ac status. The results of ChIP analyses using an anti-acetyl-Histone H3 antibody are shown. DNA sequences were quantified by real-time PCR using promoter primers. Average percentage input ± SEM (n = 3) from each experiment is plotted. *p<0.05, ***p<0.001 compared with positive-control cells.

**Figure 5:**
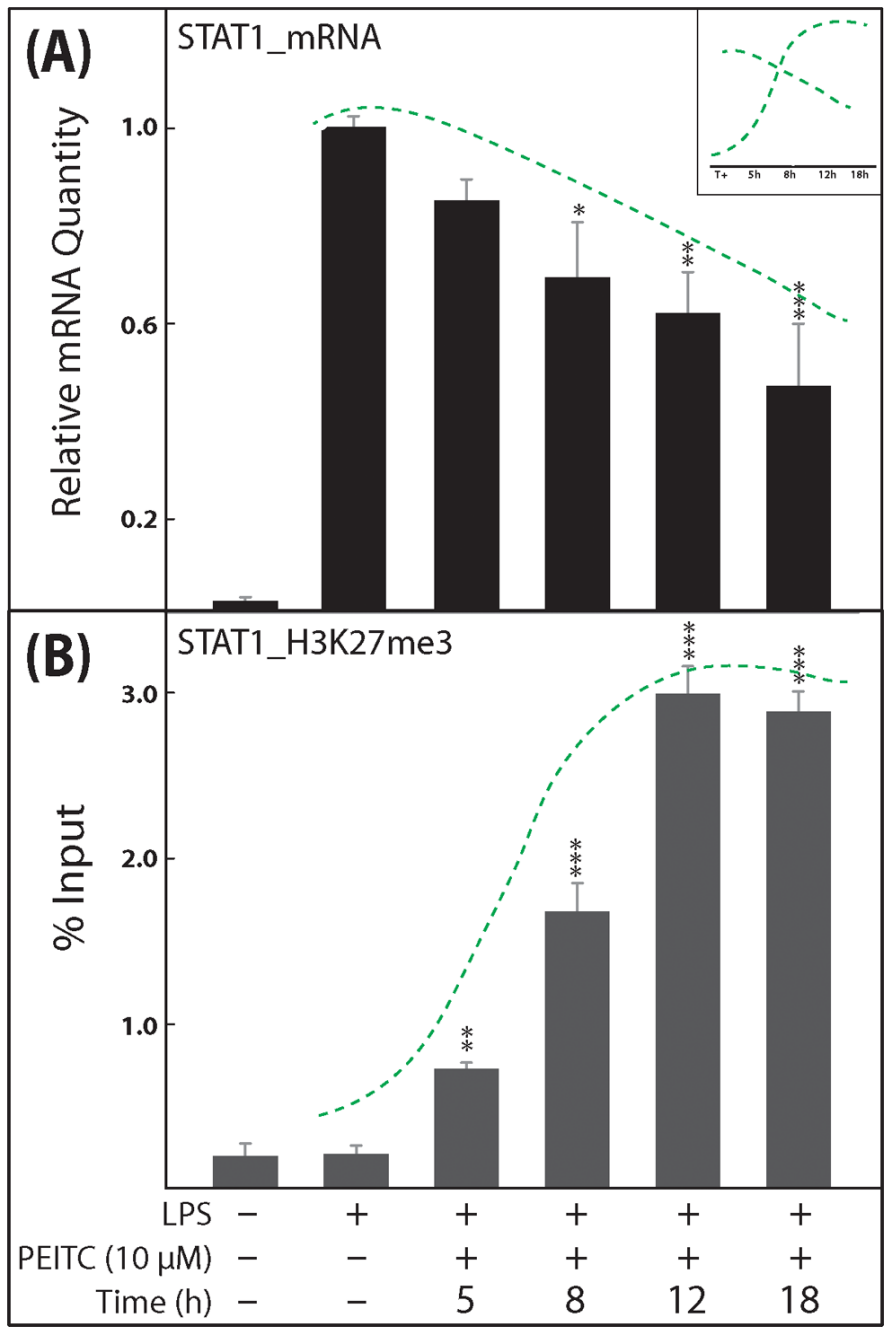
**Representative results showing the time-dependent effects of 10 µM PEITC treatment on STAT1 mRNA levels and on the H3K27me3 methylation state.** SW480 cells were treated with 10 µM PEITC at the indicated time points (A, B). The STAT1 mRNA levels were normalized to GAPDH levels and expressed as a percentage relative to positive-control cells (A). Histone H3 methylation changes at the STAT1 promoter region in SW480 cells were determined using an anti-H3K27me3 antibody for ChIP. DNA sequences were quantified by real-time PCR (B). Data points represent the mean ± SEM (n = 4) from each experiment. *p<0.05, **p<0.01, ***p<0.001 compared with positive-control cells. The green dotted lines indicate a possible inverse correlation (for repressive marks) between changes in mRNA levels and H3 modification status in the cells. The presence or absence of similar correlations for the remaining five GoI are shown in Figures S1, S2, S3, S4, S5.

**Figure 6:**
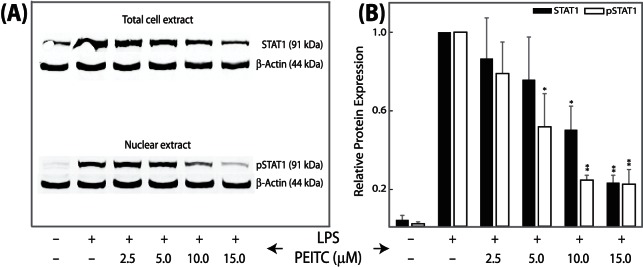
**Immunoblot analyses showing suppression of total cellular STAT1 as well as activated nuclear pSTAT1 in response to PEITC treatments in SW480 cells in a concentration-dependent manner.** (A) Immunoblot. (B) Densitometric analyses of immunoblot. Cells were pre-treated with various concentrations of PEITC for 5 h prior to 4 h LPS activation. Unstimulated cells and stimulated cells served as negative and positive controls. Band intensities were normalized to β-actin. Values are expressed as means ± SEM (n = 3) of three separate experiments. *p<0.05, **p<0.01.

A CellTiter 96 AQueous One Solution Cell Proliferation Assay kit (MTS, 3-(4,5-dimethylthiazol-2-yl)-5-(3-carboxymethoxyphenyl)-2-(4-sulfophenyl)-2H-tetrazolium, inner salt; Promega, Madison, WI) was used to determine the relative number of viable SW480 cells remaining after PEITC treatment, according to the manufacturer’s instructions. The assay was performed by treating SW480 cells with different PEITC concentrations, followed by adding 20 µl CellTiter reagent directly to culture wells, incubating for 2 h at 37°C, and then recording the absorbance at 490 nm with a BioTek Synergy H4 plate reader (BioTek, Winooski, VT). Background 490 nm absorbance was corrected by preparing a triplicate set of control wells (without cells) containing the same volumes of culture medium and CellTiter reagent as in the experimental wells. The same assay was also carried out using higher concentrations of PEITC (up to 80 µM) and exposing cells for longer time periods of up to 48 h to determine the anti-proliferation effects of PEITC in SW480 cells (Figure 1). It should be noted that even lower concentrations (above 5 µM) of PEITC treatment for longer incubation periods such as 48 h induce significant cell death and mRNA degradation (Figure 1). Concentrations of PEITC at which no significant loss of cell viability occurred (no cytotoxic effects) were selected for all further experiments (Figures 2, 3, 4, 5, 6). Hence, all gene expression assays (RNA and protein expression) and epigenetic changes reported in this manuscript were determined at the 5 h time point unless otherwise mentioned. A 40 µM exposure of PEITC for 12 h or less did not show a statistically significant loss in cell viability (Figure 1A). For time-dependent observations (Figure 5 and S1, S2, S3, S4, S5), cells with 10 µM PEITC treatment were incubated for up to 18 h, as a statistically significant loss in cell viability was not observed until that time point (Figure 1A).

### DNA Fragmentation Assay for Detection of Apoptosis

SW480 cells treated with different concentrations of PEITC were incubated for 48 h before harvesting genomic DNA using DNAzol (Invitrogen, Grand Island, NY), following the manufacturer’s protocol. DNA fragmentation was visualized using 1.5% agarose gel electrophoresis and GelRed staining (Biotium, Hayward, CA).

### Total RNA Extraction, Purification, and cDNA Synthesis

Total RNA was extracted from cells using TRIzol reagent (Invitrogen, Grand Island, NY), following the manufacturer's instructions. RNA was quantified by absorption measurements at 260 and 280 nm using the NanoDrop spectrophotometer system (NanoDrop Technologies, Wilmington, DE). RNA was then treated with DNase I (Invitrogen Inc.), following the manufacturer's guidelines to remove any traces of DNA contamination. The cDNAs were synthesized using 3 µg of RNA for each sample using the High-Capacity cDNA Reverse Transcription Kit (Applied Biosystems, Foster City, CA), following the manufacturer’s protocol. RNA extraction, purification, and cDNA synthesis were performed as previously described [13].

### Real-Time Quantitative PCR

The synthesized cDNAs were diluted four-fold. Two microliters of each diluted sample was added to 0.5 µl of gene-specific primers and 12.5 µl of Power SYBR Green PCR master mix (Applied Biosciences, Foster City, CA) and the final volume brought to 25 µl by adding sterile distilled water. PCR amplifications were performed on a MX3005P system (Roche/Stratagene) using one cycle at 50°C for 2 min, one cycle of 95°C for 10 min, 40 cycles of 15 s at 95°C and 1 min at 60°C, and a last cycle with 95°C for 1 min, 55°C for 30 s, and 95°C for 30 s, as described [13]. NTC (no template control), no-RT control, and PEITC-only controls were utilized as appropriate for quality control purposes. All samples were run in duplicate. Gene-specific, intron-spanning primers used in the current study are described in Table 1 (for PEITC concentration-dependent mRNA expression) and Table 2 (for ChIP experiments). Calculations of relative expression levels were performed using the ΔΔC_t_-method [25]. Data values are averages of at least three independent experiments and are expressed as the relative mRNA quantity with respect to a positive control, which is normalized to a value of 1.0.

**Table 1:** Primer sequences used for gene expression analyses.

Gene (accession number)	Forward	Reverse
GAPDH (NM_ 001256799.1)	5′-agccacatcgctcagacac-3′	5′-gcccaatacgaccaaatcc-3′
CASP3 (NM_004346.3)	5′-ctggttttcggtgggtgt-3′	5′-ccactgagttttcagtgttctcc-3′
CASP8 (NM_001080124.1)	5′-ggtcacttgaaccttgggaat-3′	5′-tttctgctgaagtccatcttttt-3′
CCND1 (NM_053056.2)	5′-gaagatcgtcgccacctg-3′	5′-gacctcctcctcgcacttct-3′
CD40 (NM_001250.4)	5′-ggtctcacctcgctatggtt-3′	5′-cagtgggtggttctggatg-3′
CCL2 (NM_002982.3)	5′-agtctctgccgcccttct-3′	5′-gtgactggggcattgattg-3′
IL8 (NM_000584.2)	5′-agacagcagagcacacaagc-3′	5′-atggttccttccggtggt-3′
MMP7 (NM_002423.3)	5′-ctgacatcatgattggctttg-3′	5′-atctcctccgagacctgtcc-3′
NFκB1 (NM_003998.2)	5′-accctgaccttgcctatttg-3′	5′-agctctttttcccgatctcc-3′
STAT1 (NM_007315.3)	5′-tgagttgatttctgtgtctgaagtt-3′	5′-acacctcgtcaaactcctcag-3′

Primers are shown for genes that are discussed in the paper beyond cell-screening experiments.

**Table 2:** Primer sequences used for ChIP experiments.

Gene	Forward	Reverse
p-IL8	5′-cagagacagcagagcacac-3′	5′-acggccagcttggaagtc-3′
p-STAT1	5′-gctggtcgtcactctcacaa-3′	5′-gaaagggcctacctacctcatt-3′
p-CD40	5′-gaggggaaaaccgtgagg-3′	5′-gcgaccggagagagatcc-3′
p-CCL2	5′-gtggtcagtctgggcttaatg-3′	5′-ctgctgagaccaaatgagca-3′
p-MMP7	5′-cacacagcagcatttccatc-3′	5′-tcctgtggttctccatacagg-3′
p-NFκB1	5′-ttggcaaaccccaaagag-3′	5′-ggtttcccacgatcgattt-3′

### Western Blot Analysis

For immunoblot analyses, IFNγ-primed, PEITC-treated SW480 cells were activated with LPS for 4 h and harvested using RIPA lysis buffer (20 mM Tris-HCl pH 7.5, 150 mM NaCl, 1 mM Na_2_EDTA, 1 mM EGTA, 1% NP-40, 1% sodium deoxycholate, 2.5 mM sodium pyrophosphate, 1 mM β-glycerophosphate, 1 mM Na_3_VO_4_, 1 µg/ml leupeptin). For nuclear extract preparation and protein concentration determination, the manufacturer’s (Thermo Scientific, Rockford, IL) protocols for NE-PER Nuclear Extraction Reagents and the Pierce BCA Protein Assay kit were followed. Proteins (50 µg/lane) were separated by 12% SDS-PAGE and the products were electro-transferred to polyvinyldene difluoride (PVDF) membranes (Thermo Scientific, Rockford, IL). The membranes were blocked with 5% skim milk for 1 h, washed three times in PBS, incubated with primary antibody (rabbit anti-β-actin from Santa Cruz Biotechnology and rabbit anti-STAT1 and anti-phosphorylated STAT1 [Ser727] from Millipore) overnight, washed three times in Tween-20 (0.1% in PBS), and incubated with Dylight 800 anti-rabbit secondary antibody from Li-Cor for 1 h, all at room temperature. After rinsing in Tween-20 (0.1% in PBS), blots were imaged with an Odyssey infrared imaging system (Li-Cor).

### Quantitative Chromatin Immunoprecipitation (ChIP) Analysis

Cells were plated at a density of ∼3–5×10^6^ per well in 6-well plates 18–24 h before treatment, then rinsed twice with cold PBS and harvested after treatment [26]. The ChIP assay was performed using a modified version of a previously published method [27]. Cells were suspended in ice-cold Buffer 1 (0.06 M KCL, 15 mM NaCl, 5 mM MgCl_2_, 15 mM Tris-HCl pH 7.4–7.6, 0.1 mM EGTA, 0.3 M sucrose, 180 µg aprotinin, 5 mM sodium butyrate, 0.1 mM PMSF, 0.5 mM DTT) and Buffer 2 (0.8% NP40+ Buffer 1) for 10 min on ice. Cell suspensions were added to new tubes containing Buffer 3 (0.06 M KCL, 15 mM NaCl, 5 mM MgCl_2_, 15 mM Tris-HCl pH 7.4–7.6, 0.1 mM EGTA, 1.2 M sucrose, 180 µg aprotinin, 5 mM sodium butyrate, 0.1 mM PMSF, 0.5 mM DTT). After the samples were centrifuged, the nuclei were collected and re-suspended in MNase digestion buffer (0.32 M sucrose, 50 mM Tris-HCl pH 7.4–7.6, 4 mM MgCl_2_, 1 mM CaCl_2_, 0.1 mM PMSF, 5 mM sodium butyrate), incubated in a 37°C water-bath for 6 min, and 20 µl of 0.5 M EGTA added to stop the reaction. In this way, chromatin was digested to an average DNA length of 300–800 bp. After centrifugation, the supernatant contained the first soluble fraction of chromatin, S1. The pellet was re-suspended in dialysis buffer (1 mM Tris-HCl pH 7.4–7.6, 0.2 mM EDTA, 0.2 mM PMSF, 5 mM sodium butyrate), placed on ice for 1–2 h, and centrifuged. The resulting pellet was the second soluble chromatin fraction, S2. Aliquots of the two chromatin fractions (10–20 µg S1 and 10–20 µg S2) were mixed, the volume diluted to 1 ml with ChIP incubation buffer (Abcam), and the mixture subjected to immunoprecipitation with specific antibodies, with rotation overnight at 4°C. Immunoprecipitated chromatins were extracted by using protein-A sepharose and purified by using a DNA-purifying slurry (Diagenode, Denville, NJ). An aliquot of each immunoprecipitated DNA template (100–150 ng) was used for quantitative real-time PCR analysis using sequence-specific gene promoter primers (Table 2). The PCR-amplified immunoprecipitated DNA signal was normalized to the PCR signal from non-immunoprecipitated input DNA [28]. The signals obtained by precipitation with control IgG were subtracted from the signals obtained with the specific antibodies. Calculations were based on the average of at least three independent experiments, and the results are expressed as a percentage of the input (Figures 3, 4, 5, S1, S2, S3, S4, S5).

### Statistical Analysis

The statistical significance of treatment groups was assessed by one-way ANOVA followed by Dunnett’s posthoc analysis for comparison of individual treatment group with the control. Data are expressed as means ± SEM. All experiments were repeated at least three times. A probability (*p*) value of 0.05 or less was considered to be the criterion for a significant difference.

## Results

### Effect of PEITC on Gene Expression in Human Cells

To reexamine the gene expression results in response to PEITC treatments obtained in our previous study of mouse macrophages [12], we carried out assay optimizations using three human cell lines: the human monocytic cell line THP-1 and the human colon epithelial cell lines HT-29 and SW480. Optimization included induction parameters of the inflammatory response in cells treated with LPS or LPS+IFNγ and the range of non-cytotoxic PEITC concentrations and exposure durations. Information did not exist in the literature about PEITC effects in SW480 and THP-1 cells. In the previous study [12], 21 LPS-induced genes were suppressed three-fold or more by 10 µM PEO (PEITC Essential Oil) compared with LPS activation alone in RAW 264.7 mouse macrophage cells. (PEO is PEITC obtained from *Barbarea verna* seeds [15]. Both PEO and commercially purchased PEITC are composed of >95% pure PEITC. In the current study with THP-1, HT-29, and SW480 cells, we included two additional genes, MMP7 and MMP9, for a total of 23 final genes of interest (GoI). PEITC suppressed 9, 3, and 13 out of the 23 genes in THP-1, HT-29, and SW480 cells, respectively (Table 3). The results also showed that SW480 cells were the most responsive to IFNγ-primed LPS induction among the three cell lines tested. The 13 genes down-regulated by PEITC treatment in SW480 cells represent key cellular players in inflammation and cancer. Therefore, all subsequent gene expression experiments were carried out using these 13 genes in SW480 cells. The 10 genes that were screened but were not induced/expressed in the human cell lines are not discussed further in this manuscript. Subsequent to gene expression analyses, changes in histone modifications (H3K27me3, H3K9me2, and H3-Ac) in the promoter regions of each of these 13 genes were investigated. We observed differential H3-modifications relative to controls for only six out of these 13 genes, which are discussed in detail in the manuscript. All 13 genes, whether or not H3 modifications were observed in their promoter regions in association with PEITC exposure, are listed in Table 3.

**Table 3:** Genes down-regulated (≥3-fold vs LPS activation alone) by PEITC in various human cells.

Genesymbol	Gene name	Partial Gene Ontology term(www.geneontology.org)	Responsivecell lines	% change in “S”to 10 µM PEITC	H3 changes in PEITC- treated “S”*
CCL2	Chemokine (C-C motif) ligand 2	Inflammatory response; Chemokine activity	S/T	82.43	H3K27me3
CD40	CD40 antigen	Signal transduction; Immune response;Apoptosis	S/T	49.52	H3K27me3, H3K9me2
CSF2	Colony stimulating factor 2(granulocyte -macrophage)	Immune response; Cytokine andchemokine- mediated signaling pathway	S	36.55	NT
CXCL10	Chemokine (C-X-C motif) ligand 10	Inflammatory response; Chemokine activity	S/H/T	91.63	ND
IL8	Interleukin 8	Immune response; Cytokine activity	S/H	82.43	H3K27me3, H3Ac
MMP7§	Matrix metalloproteinase 7	Zinc-dependent endopeptidases	S	36.87	H3K27me3, H3Ac
MMP9	Matrix metalloproteinase 9	Zinc-dependent endopeptidases	S/T	84.50	ND
NFκB1	Nuclear factor of kappa light chain geneenhancer in B-cells 1, p105	DNA binding; Regulation of transcription	S/T	41.98	H3K27me3
NFκBiα	Nuclear factor of kappa light chain geneenhancer in B-cells inhibitor, alpha	Nucleus; Protein binding; Cytoplasm;Regulation of cell proliferation;Protein-nucleus import, translocation	S/T	38.44	NT
REL	Reticuloendotheliosis oncogene	DNA binding; Regulation of transcription	S	28.80	ND
RELb	Avian reticuloendotheliosis viral (v-rel)oncogene related B	Transcription factor activity; Intracellular;T-helper 1 type immune reponse	S/T	66.8	ND
STAT1	Signal transducer and activatorof transcription 1	DNA binding; Regulation of transcription	S/H/T	11.28	H3K27me3
TNFαip3	Tumor necrosis factor, alpha-inducedprotein3	Apoptosis; Zinc ion binding	S/T	58.05	NT

Cell lines **SW480, S**; HT-29, H; THP-1, T.

All except MMP7 and MMP9 genes were tested in RAW macrophages in our previous report; Genes that were responsive in RAW cells but did not express/induce in any of the human cell lines are not listed here, but were previously reported as potential targets of PEITC (Dey et al., 2010).

§ Novel PEITC-mediated effect observed in the current study.

NT: Not Tested for H3 modification due to unsuccessful design of promoter primer.

ND: Tested, but none of the three H3 modifications were observed.

* Information based on 5 h incubation with PEITC in SW480 cells.

### Effect of PEITC on Colon Cancer (SW480) Cell Proliferation

Several of the selected genes observed to be modulated by PEITC in SW480 cells regulate cell proliferation during carcinogenesis (Table 3). Therefore, we investigated whether PEITC had an antagonistic effect on tumor cell proliferation, finding that PEITC attenuated viability in SW480 cells in a time- and concentration-dependent manner (Figure 1A). Interestingly, the PEITC concentrations and exposure times necessary to halt cancer cell multiplication are higher and longer, respectively, than those necessary to induce changes in chromatin chemistry (Figures 3, 4, 5, S1, S2, S3, S4, S5) and gene expression (Figures 2, 6, S1, S2, S3, S4, S5). For concentrations below 80 µM, at least a 24 h exposure was necessary to significantly induce cancer cell death (Figure 1). For most of the remaining experiments (Figures 2, 3, 4, and 6), we used a PEITC exposure of 5 h at or below 15 µM. In Figures 5 and S1, S2, S3, S4, S5, 10 µM PEITC was used at various time points in SW480 cells for up to 18 h of PEITC exposure time. A 10 µM concentration of PEITC significantly affected cell viability at or beyond 24 h (Figure 1A), and a DNA fragmentation assay (Figure 1B) revealed that the antiproliferative effects of PEITC may be due to apoptotic up-regulation as also evidenced by higher expression of caspase 3 and 8 (Figure 1C). The absence of significant changes in the cell cycle protein CyclinD1 (CCND1) indicates that PEITC does not directly inhibit the cell cycle (Figure 1C).

### Modulation of Chemokines by PEITC in Human Colon Cells

Chemokines play a major role in the maintenance of inflammatory processes in the large bowel. The production of chemokines within the intestine establishes a chemotactic gradient capable of increasing the migration of monocytes/macrophages, granulocytes, and lymphocytes from the bloodstream through the endothelium into both the mucosa and submucosa during chronic inflammatory bowel diseases (IBD) [29]. Previously, we observed that PEITC reduced inflammation, depletion of goblet cells, and infiltration of inflammatory cells in mouse mucosa and submucosa [12]. In the current study, concentration-dependent PEITC-mediated attenuation of mRNA levels of four proinflammatory chemokines/cytokines (CCL2, CSF2, CXCL10, and IL8) was observed in SW480 cells (Table 3, Figure 2). Individual ChIP experiments on these chemokine promoters revealed hyper-trimethylation of H3 at lysine 27 (increased H3K27me3 states) surrounding the promoter regions of IL8 and CCL2 and an additional decreased acetylation surrounding the promoter regions of IL8 associated with 10 µM PEITC exposure (Tables 3–4, Figures 3A and 4). However, a time-dependent inverse correlation was observed for H3K27me3 (repressive mark) but not H3-Ac with IL8 mRNA expression levels (Table 4, Figure S5). In the case of CCL2, mRNA levels and H3K27me3 states were observed to vary together, ruling out a potential causal relationship (Table 4, Figure S4). Although polycomb repressive complex 2 (PRC2)-catalyzing H3K27 trimethylation is involved in cytokine gene reprogramming in response to inflammatory stimuli [30], [31], the apparent selectivity of PEITC in the context of histone modifications surrounding the promoter regions of the targeted chemokines/cytokines leads us to believe that PEITC is unlikely to directly affect the PRC2.

**Table 4:** Time-dependent correlation between mRNA expression and H3 modifications.

Gene Name	Effects	10 µM PEITC treatment at four time points: 5, 8, 12, 18 h
		Observation summary (significance compared with control)
STAT1	Relative mRNA H3K27me3 (%)	Levels decreased in a time-dependent manner up to 12 h Levels increased in a time-dependent manner up to 12 h; correlate with mRNA expression *(this data is shown as a representative histogram in* *Figure 5* *in main text; while remaining data presented in this table are shown as Figures S1, S2, S3, S4, S5)*
NFκB1	Relative mRNA	Suppression of mRNA only at 5 h; for 8 h onward, no significant suppression
	H3K27me3 (%)	With increase of mRNA levels 8h onward, H3K27me3 levels decreased and lost significance; correlates with mRNA expression *(Figure S1)*
MMP7	Relative mRNA	Levels decreased in a time-dependent manner up to 18 h
	H3K27me3 (%)	No correlation with mRNA levels except at 5 h. H3K27me3 and mRNA levels both decreased at later time points *(Figure S2)*
	H3Ac (%)	Levels decreased in a time-dependent manner up to 18 h; correlate with mRNA expression *(Figure S2)*
CCL2	Relative mRNA	Expression levels significantly low at all time points;
	H3K27me3 (%)	Correlation with mRNA unlikely *(Figure S4)*
CD40	Relative mRNA	Expression levels significantly low at all time points;
	H3K27me3 (%)	Changes do not correlate with mRNA levels over time *(Figure S3)*
	H3K9me2 (%)	Changes do not correlate with mRNA levels over time *(Figure S3)*
IL8	Relative mRNA	Suppression of mRNA only at 5 h; for 8 h onward, no significant suppression
	H3K27me3 (%)	With increase of mRNA levels, H3K27me3 level decreased, but remained significantly higher than control; partial correlation with mRNA levels likely *(Figure S5)*
	H3Ac (%)	Acetylation levels remained low even after mRNA suppression was lost at later time points. No time-dependent correlation with mRNA levels *(Figure S5)*

Effects: mRNA levels are normalized to GAPDH levels and quantified relative to positive control; ChIP values are determined as the percentage of input; *p<0.05 is considered significant.

H3K27me3: trimethylation of histone H3 at lysine 27; H3K9me2: dimethylation of histone H3 at lysine 9; both marks were expected to have an inverse correlation with mRNA expression; H3Ac: acetylation of histone H3; this mark was expected to have a direct correlation with mRNA expression; all six listed genes were tested for changes in H3K27me3, H3k9me2, and H3-Ac changes in their promoter regions. Only statistically significant differential observations relative to positive control (LPS-induced but not treated with PEITC) are noted in the table and discussed in the manuscript.

### PEITC Exposure Perturbs Transcription Factor Activities in Human Colon Cells

The NFκBs and STATs are two important families of transcription factors activated in response to a variety of stimuli to regulate multiple cellular processes, including the immune response and carcinogenesis. The NFκBs and STATs have distinct as well as synergistic effects on downstream effector gene induction [32]. The role of NFκB in IBD [33] as well as the effect of PEITC on NFκB activity [34], [35], [36], [37] are well studied. Here we observed PEITC-mediated down- regulation of various NFκB family members, namely NFκB1, NFκBiα, and REL proteins, which was not previously reported for SW480 cells (Table 3, Figure 2). Intriguingly, a time-dependent increase in the H3K27me3 state was associated with a time-dependent decrease in NFκB1 expression in PEITC-exposed cells, suggesting potential epigenetic regulation of NFκB1 that merits future validation (Table 4, Figures 3A, S1). Additionally, STAT proteins are dormant cytoplasmic transcription factors that become activated after phosphorylation by kinases in response to various stimuli, including cytokines, growth factors, and LPS [32]. The activated proteins migrate into the nucleus and bind to specific promoter elements to regulate gene expression [38]. An increase in STAT1 expression and activation in human ulcerative colitis (UC) was reported [22], and UC patients are at higher risk of colon cancer [22]. We had earlier reported a novel PEITC-mediated attenuation of activated STAT1 in mouse cells [12]. Here we observed a PEITC-mediated down-regulation of STAT1 mRNA in all three human cell lines (Table 3, Figures 2, 5). Changes in STAT1 mRNA expression also correlated with time-dependent changes in H3K27me3 levels surrounding the promoter region of STAT1 in SW480 cells (Figures 3A and 5). Since STAT1 is a transcription factor with the potential to regulate numerous downstream effector genes and since, until now, the relationship between STAT1 expression and activation in response to PEITC exposure has been relatively unexplored (aside from our own observations), we delved deeper into the nature of this relationship. The activated form of STAT1 (phosphorylated at Serine 727) was not detected at all in the nuclear extracts (Figure 6) until after activation with LPS in IFNγ-primed SW480 cells. Once activated, an increase in STAT1 and pSTAT1 proteins was observed (Figure 6), both of which were attenuated by PEITC in a concentration-dependent manner. In our mRNA detection experiments, the levels of STAT1 were lowered by PEITC in both a time- (Figure 5) and concentration-dependent (Figure 2) manner that was inversely correlated (inset, Figure 5) with H3K27me3 levels (Figures 3A and 5). However, even after an 18 h exposure to 10 µM PEITC, the levels of STAT1 mRNA did not revert to unstimulated levels (Figure 5). Also, changes in H3K9me2 or acetylation levels surrounding the STAT1 promoter were not observed in response to PEITC treatments (Table 4), indicating that the effects of PEITC on chromatin chemistry are likely directed in a highly gene promoter-specific manner.

### Effect of PEITC on MMPs in Human Cancer Cells

MMPs are considered to be the predominant proteases in the pathogenesis of mucosal ulcerations associated with IBD. MMP7 expression in inflammatory and endothelial cells suggests that it has a role in both colitis-associated neoangiogenesis and inflammatory changes [39]. In many malignancies, including colorectal cancers, MMP7 and MMP9 are overexpressed and play a role in cancer progression by enhancing metastatic potential and tumor invasion [40], [41]. While induction of both MMP7 and MMP9 were reduced by PEITC, possible epigenetic control of such repression was only observed for MMP7 (Table 3). The promoter of MMP7 showed H3 hypermethylation at lysine 27 and hypoacetylation of H3 in PEITC-treated cells compared with cells not exposed to PEITC (Figures 3A, 4). Interestingly, time dependent correlation of H3 modification and MMP7 expression was only observed for H3-Ac state (activation mark, hence, directly correlated) and not for the H3K27me3 state (Table 4, Figure S2). In our previous report, we did not test the effects of PEITC treatment on either of the MMP genes. However given their key role in the pathogenesis of IBD and in development of colon cancer, we included these two genes in our current investigation. To our knowledge, mRNA attenuation of MMP7 by PEITC as well as PEITC-exposure-associated changes in chromatin decorations on the MMP7 promoter have not been reported previously.

## Discussion

Epigenetic changes, although can be heritable, are potentially reversible and may affect genomic stability and gene expression. In recent years, great strides have been made in understanding the epigenetic mechanisms that determine which genes can be turned on and off and the implications for human health and disease. Importantly, we now know that dietary factors and specific nutrients can modulate epigenetic signatures (decorations) and, therefore, susceptibility to disease [42]. Targeting the epigenome, including the use of HDAC and DNA methyltransferase (DNMT) inhibitors, is an evolving strategy for cancer chemoprevention and has shown promise in cancer clinical trials [43]. However, it is not known whether such a broad targeting strategy against the cancer epigenome (in cancer cells) also has pleiotropic effects on cellular pathways in normal cells. Here we present novel PEITC-exposure-associated, potentially selective site- and promoter-specific regulation of epigenetic events in cancer cells that may further validate the health-protective utility of this chemopreventive/chemotherapeutic agent.

PEITC and other isothiocyanates are capable of inhibiting the development of cancer in experimental models through multiple pathways, including, but not limited to, induction of carcinogen-detoxifying phase 2 enzymes, induction of apoptosis, and inhibition of cell cycle progression [44]. Previously, we had reported that orally administered PEITC is pharmacologically active in remitting acute and chronic bowel inflammation in mouse UC models [12]. The biological activity of PEITC is related to the inhibition of proinflammatory cytokines produced in the inflamed gut. We subsequently elucidated potential novel biomarkers associated with PEITC exposure in mouse macrophage cells [12]. We observed that 21 genes with cellular functions related to inflammation, apoptosis, cell cycle regulation, proliferation, chemokine activity, and transcriptional regulation were modulated in response to PEITC treatment [12]. Since persistent inflammation in the colon is a major risk factor for the development of colon carcinogenesis, we subsequently hypothesized that the ability of PEITC to ameliorate the functions of inflammatory and cell death mediators could be responsible for its previously observed chemoprotective role in the colon [23]. The current study reports that PEITC attenuates the proliferation of human colon cancer cells (SW480) in a concentration- and time-dependent manner (Figure 1A), likely mediated by a caspase-dependent apoptotic pathway. Concentration- dependent up-regulation of an initiator caspase (caspase 8) followed by an effector caspase (caspase 3, Figure 1C), which corresponds to DNA fragmentation in the cells (Figure 1B), in response to PEITC was significant, given the key role of caspases in apoptosis [45]. We also demonstrate novel and dynamic H3-site-specific chemical changes associated with PEITC-exposure in the promoters of the GoIs (Figures 3, 4, 5, S1, S2, S3, S4, S5, Table 3), whose expression levels were also suppressed in the presence of PEITC (Figure 2, 6). The changes in H3 decorations are observed to be gene-specific, that is, a potential correlation between changes in histone modification and mRNA expression over time was not uniform across the GoIs in SW480 cells (Table 4, Figures 5, S1, S2, S3, S4, S5). To the best of our knowledge, the biological effects of PEITC have not been previously reported in SW480 cells.

In the current study, we focused our investigation on three very well-characterized histone modifications. Site-specific alteration of chromatin is a key signalling event in dynamic regulation and maintenance of the genome. Recent high-resolution profiling of histone methylation in the human genome showed increased H3K9me2 and H3K27me3 levels at the transcriptional start sites of silent/repressed genes compared with active genes [46], [47], [48], [49], [50]. A third histone mark that is directly correlated with gene expression is lysine acetylation (H3-Ac) [51], [52]. Deacetylation of sites on each of the H3, H4, and H2B histones are all associated with chromatin condensation and subsequent gene repression. For convenience, we chose the anti-acetyl-H3 antibody to keep our analysis specific to histone H3.

Altogether, we examined the effects of PEITC on 23 genes in three human cell lines representing the monocytic lineage (THP-1) as well as colon cancer-derived epithelial cell types (HT-29 and SW480, Table 3, Figure 2). Ten out of the 23 genes that were modulated in mouse macrophage cells [12] did not induce/express in response to LPS treatments in the human cell lines. Cell line-specific variation in LPS responsiveness in vitro is not uncommon in the scientific literature. Therefore, only results for the 13 genes are shown in Table 3. Out of the three cell types tested, the SW480 cells were most responsive to LPS-induction and PEITC-mediated suppression of target genes (Table 3). Since a fundamental association between histone modification levels and tumor aggressiveness has recently been proposed [7], three repressive histone marks (H3K27me3, H3K9me2, and H3-deactylation) were subsequently investigated in each of the 13 genes that were down-regulated by PEITC in SW480 cells (Table 3). Out of these 13 genes (Table 3) that were investigated for PEITC-exposure-associated changes in H3 decorations, only six (Figure 2, Table 4) showed significant changes at 10 µM PEITC exposure for 5 h. Three of these six genes had more than one type of change (Figures 2, 3, 4). All of these six genes (STAT1, NFκB1, MMP7, CD40, CCL2, and IL8) were further examined for time-dependent correlation of H3 modification states with mRNA levels (Table 4). The correlation for STAT1 is shown in the main text as a representative gene (Figure 5), while the remaining five are presented as supplementary results (Figures S1, S2, S3, S4, S5). Interestingly, an apparent time-dependent causal relationship was selectively observed for four of the six genes (Table 4, Figures 5, S1, S2, S5, correlation shown with green dotted lines; correlation can be direct or inverse depending on activation or repressive mark). H3K27me3 (repressive mark) states were inversely correlated with STAT1, NFκB1, and IL8, and H3-Ac (activation mark) levels were positively correlated with MMP7. For CD40 and CCL2, such correlations were not observed (the absence of correlation is shown with red dotted lines, Figures S3, S4). Presence as well as absence of correlation between gene expression and histone modifications indicates a possible selective gene-specific function of PEITC. A number of possible scenarios that might contribute to this are discussed below.

Three distinct classes of protein factors are responsible for the maintenance and interpretation of the post-translational modification (PTM) marks of the chromatin machinery that determine the epigenetic landscape of the cell. These are writers (enzymes adding marks), readers (protein modules binding to histone marks), and erasers (enzymes removing/editing marks). In human, writers catalyzing histone lysine methylations are site-specific HMTs (one for H3K27 and around six for H3K9), while the methyl-removing erasers are site-specific demethylases (three for each of H3K9 and H3K27). Similarly, writers and erasers of acetylation marks are site-specific histone acetyl transferases (HATs, around eight of which are known to date) and HDACs (about eleven known), respectively. In the context of gene suppression, gene-activating chromatin marks (e.g., H3-Ac) are expected to decrease while repressive marks (e.g., H3K27me3 and H3K9me2) are expected to increase at gene promoters.

The apparent selectivity of PEITC-exposure-associated, time-dependent H3K27me3 modifications surrounding the promoter regions of the GoIs (inverse correlation for STAT1, NFκB1, and IL8 with no correlation for CD40, CCL2, and MMP7) leads us to believe that PEITC may not directly affect EZH2 protein (writer), the HMT component of the PRC2 in SW480 cells. PEITC likely promotes protein–protein interactions such that the PRC2 complex (or other enzyme complexes) may be recruited in the vicinity of promoter-specific, DNA-binding transcription factors. Conversely, it is also possible that PEITC disrupts protein–protein interactions such that the demethylases are preferentially excluded in the vicinity of the promoter-specific, DNA-binding transcription factors, leading to changes in methyl marks. It is also possible that a certain type of change, potentially resulting from PEITC exposure, only occurs for genes with consensus sequences or transcription factor binding commonality within their promoters. These preferential writer/eraser localization possibilities will be explored in future and reported elsewhere. Additionally, expression levels of the writer/eraser enzymes may also influence enzyme localizations, and such a possibility is currently being examined. Hence, when a time-dependent correlation is present, investigating promoter binding (localization), expression levels, and enzymatic activity/inhibition of the site-specific writer/eraser enzymes for each of the reported GoI associated with PEITC exposure may confirm or disconfirm the underlying molecular mechanisms explaining the cause-and-effect relationship of the observed changes. It is also possible that the effect of PEITC on the epigenetic signature is cell-type dependent, which may explain why, in an earlier report, PEITC functioned as a HDAC inhibitor to activate the p21 gene in prostate cancer cells [53], while in our observations, chromatin and gene expression changes associated with PEITC exposure in colon cancer cells were repressive in nature.

The absence of an observed correlation between RNA expression and repressive histone modifications may also suggest a number of possibilities that are worth investigating in future such as: (i) demethylation of activation marks like H3K4me3 and H3K36me3 (not investigated in the current work), which may lower mRNA levels, (ii) cross talk with adjacent histone marks, i.e., combinatorial effects of multiple epigenetic marks including CpG methylation, (iii) ability and/or inability of a ChIP antibody to discriminate between a single histone mark when alone or in combination with adjacent marks (e.g., H3K27me3 vs H3K27me3S28ph), (iv) the role of micro-RNA in post-transcriptional suppression of mRNA, which is independent of histone modifications.

To summarize, PEITC down-regulated multiple genes representing key cellular functions related to the immune response and cell proliferation in human colon cancer cells. These results included the novel observation of suppression of MMP7 and other genes by PEITC in SW480 cells. Cancer pathogenesis is complex and often characterized by abnormalities in various checkpoints and oncogenic pathways, and cancer cells have altered signaling networks due to multiple alterations in gene expression. Overreliance on single-targeted interventions to treat or prevent disease has impeded the drug discovery process over the past several decades [3]. The robustness of biological systems to bypass intervention at one checkpoint necessitates interventions that target biomarkers representing multiple signaling pathways, which also applies to potential dietary chemopreventive agents [54] such as PEITC. Also, the discovery of novel biomarker(s) associated with PEITC exposure and activity is critical for its clinical development. Chemoprevention trials, with cancer incidence as the primary endpoint, are expensive and time-consuming, as they typically require thousands of human subjects, coordinated multi-institutional efforts, and years of follow-up to draw meaningful conclusions. Therefore, smaller cohort-specific pilot biomarker-modulation studies could provide molecular endpoints and hence rational justification for larger cancer prevention trials. We believe that the information from this study contributes to the knowledge base regarding the possible prognostic relevance of epigenetic signatures in cancer cells and the likely prediction of therapeutic responses based on these signatures. In addition, the role of chromatin chemistry is now appreciated beyond clinical oncology [6], such as in stress response [55], aging [56], behavior, and addiction [57]. Chromatin and also chromatin-interacting proteins have recently emerged as promising drug targets [58], [59], [60]. Therefore, the current work presents an encouraging case for the chromatin context in diet–gene interaction studies, which can potentially lead to new directions in “diet for human health” research beyond the field of cancer biology.

## Supporting Information

Figure S1 **Time-dependent effect of 10 µM PEITC treatment on NFκB1 mRNA levels and on the H3K27me3 methylation state.** SW480 cells were treated with 10 µM PEITC at the indicated time points (A, B). NFκB1 mRNA levels were normalized to GAPDH levels and expressed as a percentage relative to positive-control cells (A). Histone H3 methylation changes at the NFκB1 promoter region in SW480 cells were determined using anti-H3K27me3 antibody for ChIP. DNA sequences were quantified by real-time PCR (B). Data points represent the mean ± SEM (n = 4) from each experiment. *p<0.05, **p<0.01, ***p<0.001 compared with positive-control cells. The dotted green lines indicate a possible inverse correlation between changes in mRNA levels and H3 modification status in the cells.
(TIF)

Figure S2 **Time-dependent effect of 10 µM PEITC treatment on MMP7 mRNA levels, H3K27me3 methylation, and H3Ac acetylation states.** (i) The MMP7 mRNA levels were normalized to GAPDH levels and expressed as a percentage relative to positive-control cells. (ii) Histone H3K27 trimethylation changes and (iii) Histone H3 acetylation changes at the MMP7 promoter region in SW480 cells were determined using anti-H3K27me3 and anti-H3Ac antibodies for ChIP. DNA sequences were quantified by real-time PCR. Data points represent the mean ± SEM from each experiment. *p<0.05, ***p<0.001 compared with positive-control cells. The dotted green lines indicate the presence of a correlation (negative/inverse for methylation marks and positive/direct for acetylation marks) while dotted red lines indicate the absence of such a correlation between changes in mRNA levels and H3 modification states in the cells.
(TIF)

Figure S3 **Time-dependent effect of 10-µM PEITC treatment on CD40 mRNA levels and on H3K27me3 and H3K9me2 methylation states.** (i) The CD40 mRNA levels were normalized to GAPDH levels and expressed as a percentage relative to positive-control cells. (ii) Histone H3K27 trimethylation changes. (iii) Histone H3K9 dimethylation changes at the CD40 promoter region in SW480 cells was determined using anti-H3K27me3 and anti-H3K9me2 antibodies for ChIP. DNA sequences were quantified by real-time PCR. Data points represent the mean ± SEM from each experiment. **p<0.01, ***p<0.001 compared with positive-control cells. The dotted red lines indicate the absence of possible inverse correlations between changes in mRNA levels and H3 modification status in the cells.
(TIF)

Figure S4 **Time-dependent effect of 10-µM PEITC treatment on CCL2 mRNA levels and on the H3K27me3 methylation state.** (i) The CCL2 mRNA levels were normalized to GAPDH levels and expressed as a percentage relative to positive-control cells. (ii) Histone H3K27 trimethylation changes at the CCL2 promoter region in SW480 cells was determined using anti-H3K27me3 antibody for ChIP. DNA sequences were quantified by real-time PCR. Data points represent the mean ± SEM from each experiment. *p<0.05, **p<0.01, ***p<0.001 compared with positive-control cells. The dotted red lines indicate the absence of a possible inverse correlation between changes in mRNA levels and H3 modification status in the cells.
(TIF)

Figure S5 **Time-dependent effect of 10-µM PEITC treatment on IL8 mRNA levels and on H3K27me3 methylation and H3Ac acetylation states.** (i) The IL8 mRNA levels were normalized to GAPDH levels and expressed as a percentage relative to positive control cells. (ii) Histone H3K27 trimethylation changes and (iii) Histone H3 acetylation changes at the IL8 promoter region in SW480 cells were determined using anti-H3K27me3 and anti-H3Ac antibodies for ChIP. DNA sequences were quantified by real-time PCR. Data points represent the mean ± SEM from each experiment. *p<0.05, **p<0.01, ***p<0.001 compared with positive-control cells. The dotted green lines indicate the presence of an observed inverse correlation while dotted red lines indicate the absence of a direct correlation between changes in mRNA levels and H3 modification states in the cells.
(TIF)
