# Supplementary figures and images for: Phenethylisothiocyanate Alters Site- and Promoter-Specific Histone Tail Modifications in Cancer Cells
Source: PLoS One. 2013 May 28;8(5):e64535. doi: 10.1371/journal.pone.0064535 (PMC3665791; doi:10.1371/journal.pone.0064535)

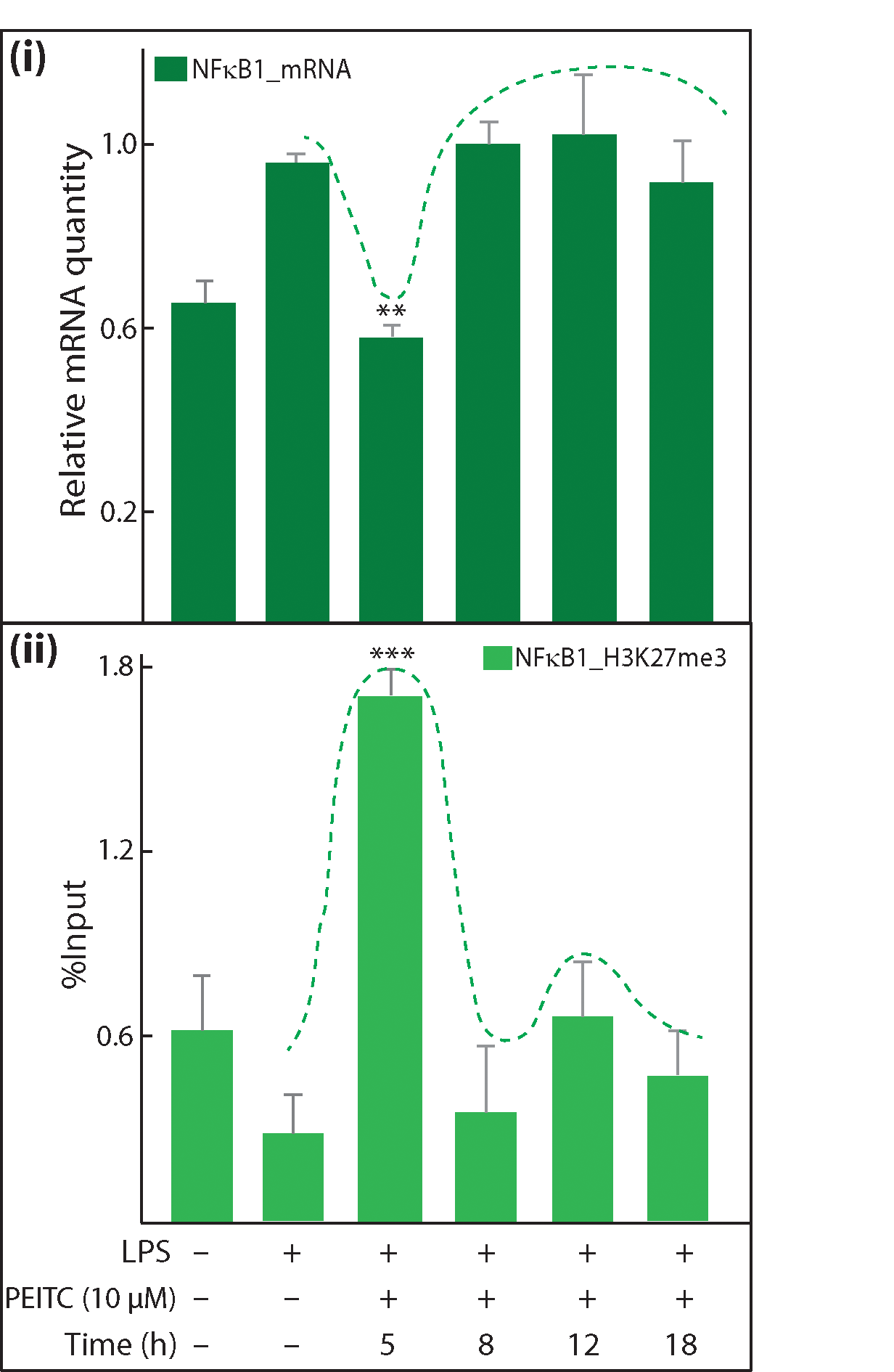

Supplement: Figure S1 — Time-dependent effect of 10 µM PEITC treatment on NFκB1 mRNA levels and on the H3K27me3 methylation state. SW480 cells were treated with 10 µM PEITC at the indicated time points (A, B). NFκB1 mRNA levels were normalized to GAPDH levels and expressed as a percentage relative to positive-control cells (A). Histone H3 methylation changes at the NFκB1 promoter region in SW480 cells were determined using anti-H3K27me3 antibody for ChIP. DNA sequences were quantified by real-time PCR (B). Data points represent the mean ± SEM (n = 4) from each experiment. *p<0.05, **p<0.01, ***p<0.001 compared with positive-control cells. The dotted green lines indicate a possible inverse correlation between changes in mRNA levels and H3 modification status in the cells. (TIF) [file pone.0064535.s001.tif]

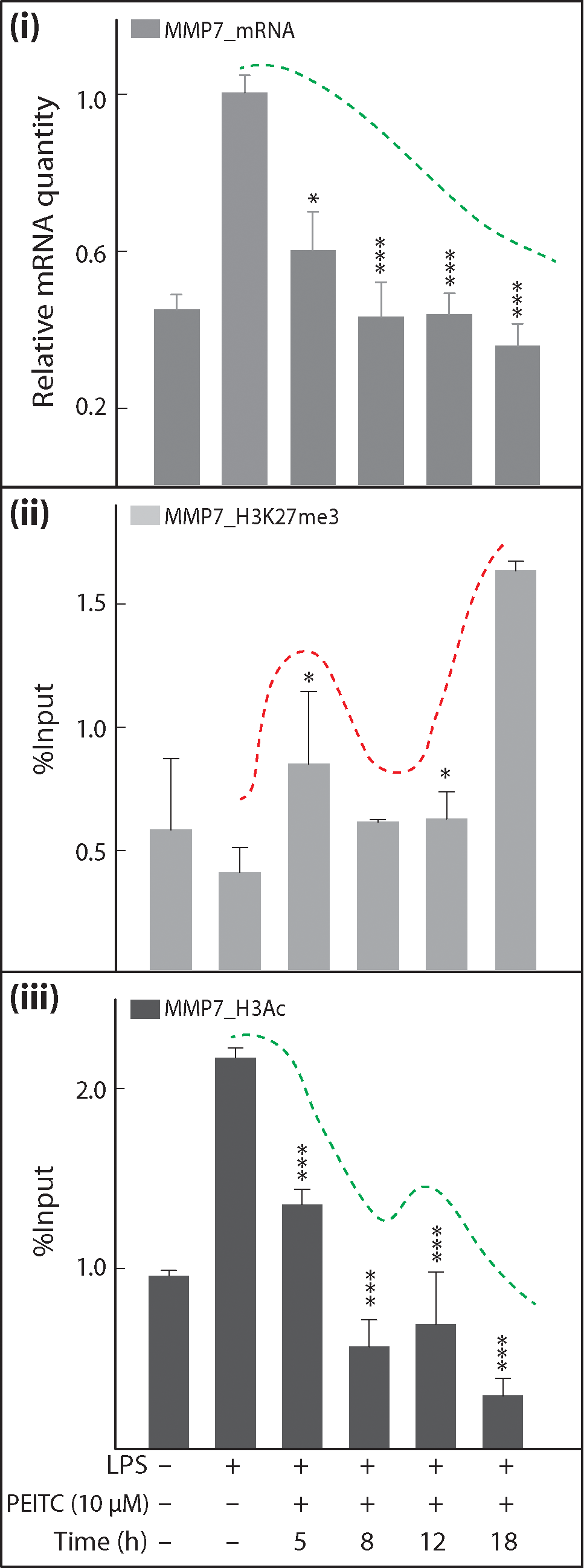

Supplement: Figure S2 — Time-dependent effect of 10 µM PEITC treatment on MMP7 mRNA levels, H3K27me3 methylation, and H3Ac acetylation states. (i) The MMP7 mRNA levels were normalized to GAPDH levels and expressed as a percentage relative to positive-control cells. (ii) Histone H3K27 trimethylation changes and (iii) Histone H3 acetylation changes at the MMP7 promoter region in SW480 cells were determined using anti-H3K27me3 and anti-H3Ac antibodies for ChIP. DNA sequences were quantified by real-time PCR. Data points represent the mean ± SEM from each experiment. *p<0.05, ***p<0.001 compared with positive-control cells. The dotted green lines indicate the presence of a correlation (negative/inverse for methylation marks and positive/direct for acetylation marks) while dotted red lines indicate the absence of such a correlation between changes in mRNA levels and H3 modification states in the cells. (TIF) [file pone.0064535.s002.tif]

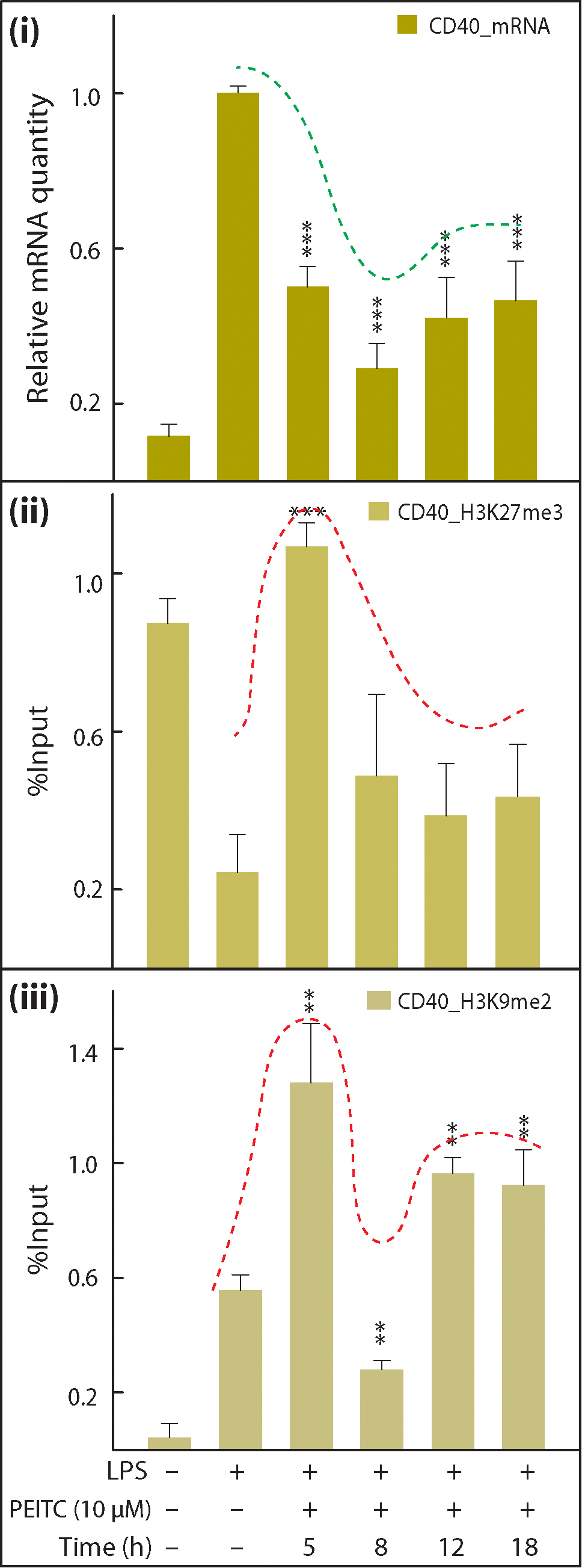

Supplement: Figure S3 — Time-dependent effect of 10-µM PEITC treatment on CD40 mRNA levels and on H3K27me3 and H3K9me2 methylation states. (i) The CD40 mRNA levels were normalized to GAPDH levels and expressed as a percentage relative to positive-control cells. (ii) Histone H3K27 trimethylation changes. (iii) Histone H3K9 dimethylation changes at the CD40 promoter region in SW480 cells was determined using anti-H3K27me3 and anti-H3K9me2 antibodies for ChIP. DNA sequences were quantified by real-time PCR. Data points represent the mean ± SEM from each experiment. **p<0.01, ***p<0.001 compared with positive-control cells. The dotted red lines indicate the absence of possible inverse correlations between changes in mRNA levels and H3 modification status in the cells. (TIF) [file pone.0064535.s003.tif]

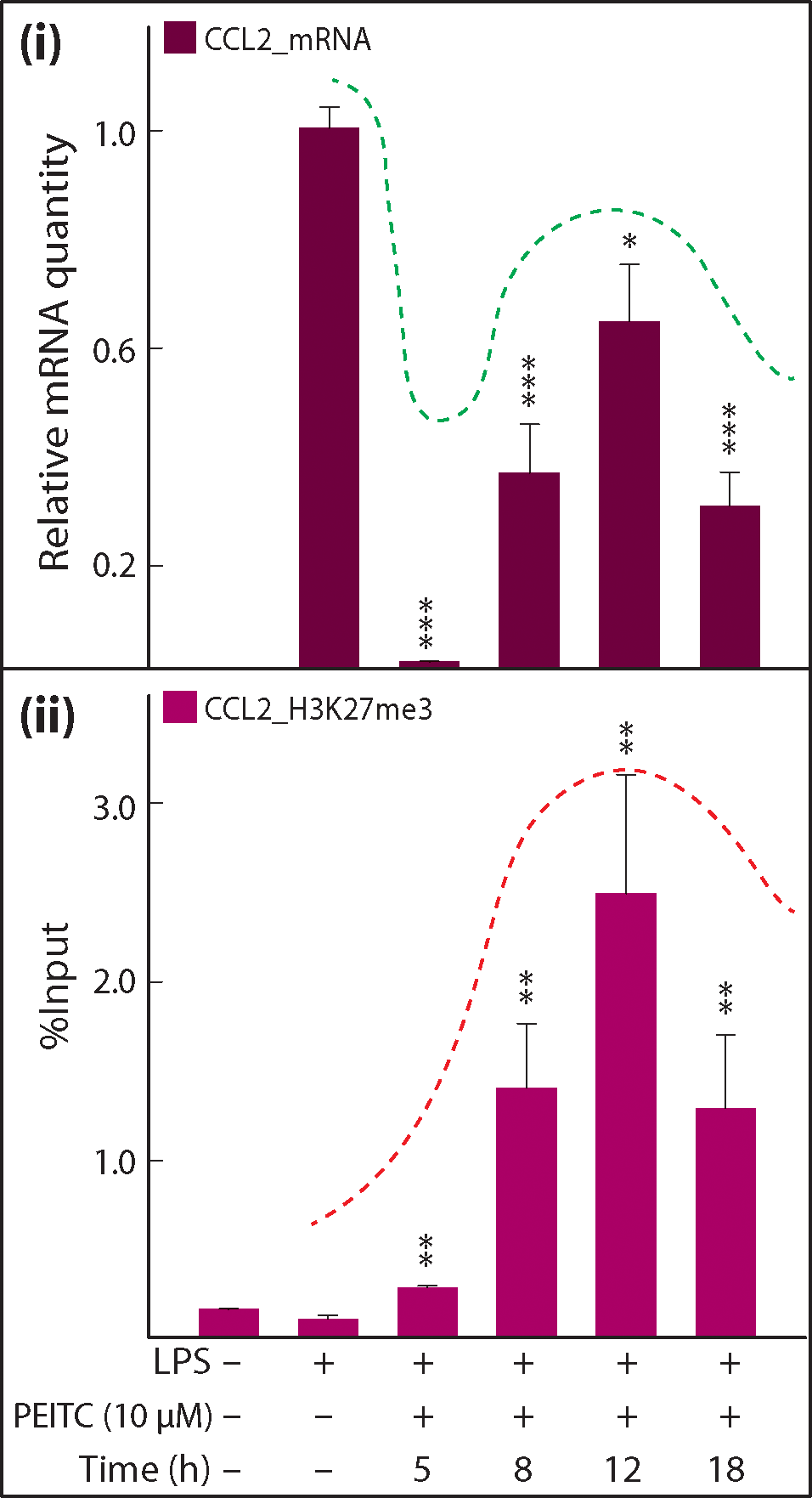

Supplement: Figure S4 — Time-dependent effect of 10-µM PEITC treatment on CCL2 mRNA levels and on the H3K27me3 methylation state. (i) The CCL2 mRNA levels were normalized to GAPDH levels and expressed as a percentage relative to positive-control cells. (ii) Histone H3K27 trimethylation changes at the CCL2 promoter region in SW480 cells was determined using anti-H3K27me3 antibody for ChIP. DNA sequences were quantified by real-time PCR. Data points represent the mean ± SEM from each experiment. *p<0.05, **p<0.01, ***p<0.001 compared with positive-control cells. The dotted red lines indicate the absence of a possible inverse correlation between changes in mRNA levels and H3 modification status in the cells. (TIF) [file pone.0064535.s004.tif]

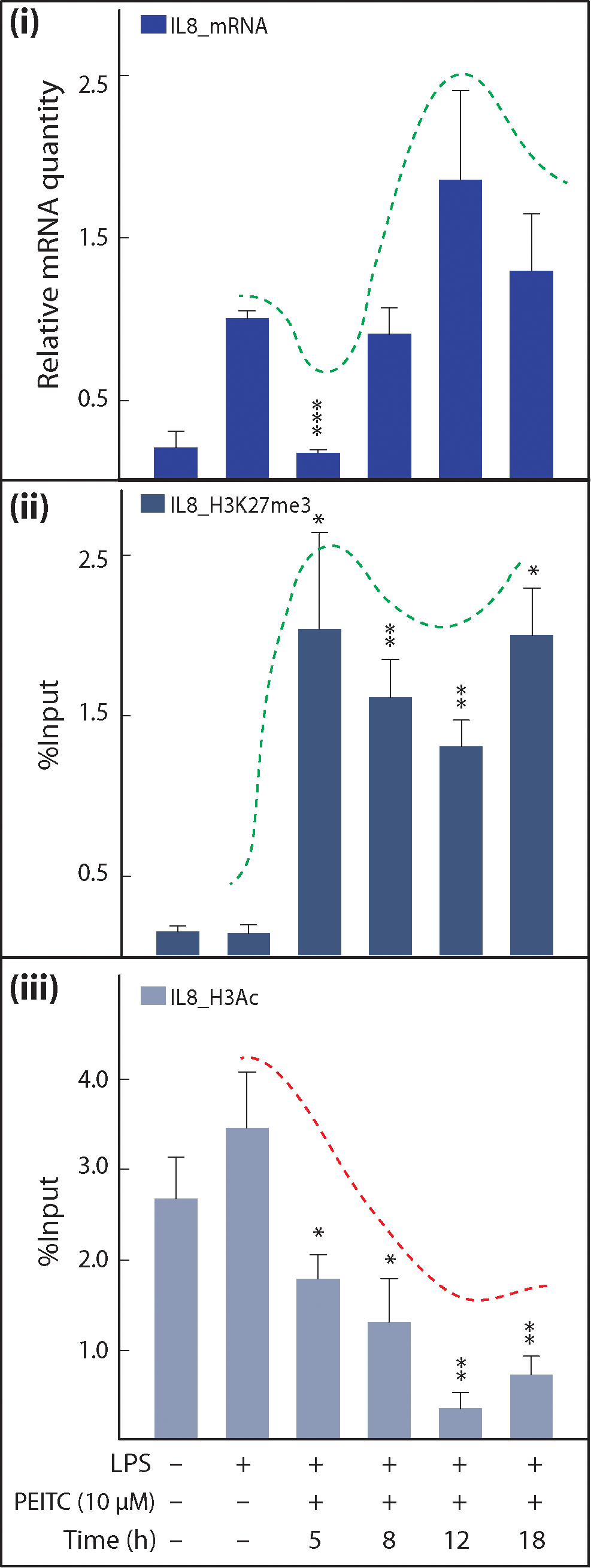

Supplement: Figure S5 — Time-dependent effect of 10-µM PEITC treatment on IL8 mRNA levels and on H3K27me3 methylation and H3Ac acetylation states. (i) The IL8 mRNA levels were normalized to GAPDH levels and expressed as a percentage relative to positive control cells. (ii) Histone H3K27 trimethylation changes and (iii) Histone H3 acetylation changes at the IL8 promoter region in SW480 cells were determined using anti-H3K27me3 and anti-H3Ac antibodies for ChIP. DNA sequences were quantified by real-time PCR. Data points represent the mean ± SEM from each experiment. *p<0.05, **p<0.01, ***p<0.001 compared with positive-control cells. The dotted green lines indicate the presence of an observed inverse correlation while dotted red lines indicate the absence of a direct correlation between changes in mRNA levels and H3 modification states in the cells. (TIF) [file pone.0064535.s005.tif]
